# The Right Hand Draws the Trees, But the Left Draws the Forest?

**DOI:** 10.3233/BEN-2008-0209

**Published:** 2009-06-01

**Authors:** Jonathan T. Kleinman, Amitabh Gupta

**Affiliations:** ^1^Department of NeurologyJohns Hopkins School of MedicineBaltimoreMDUSA; ^2^Stanford University School of MedicineCAUSA; ^3^Department of NeurologyThe Johns Hopkins HospitalBaltimoreMDUSA; ^4^Department of NeurologyUniversity of TorontoTorontoONCanada

**Keywords:** Visuospatial processing, double dissociation, corpus callosum

## Abstract

Spatial processing is lateralized: the right hemisphere is optimized for perceiving global aspects of space (“seeing the forest”), while the left hemisphere specializes in perceiving local aspects of space (“seeing the trees”). However, less is known about how the information is shared across the hemispheres and which areas within the corpus callosum are required for transferring and integrating visuospatial information. Here, we report a 60 year old woman with a mass lesion in the splenium of the corpus callosum who demonstrated visuospatial processing deficits that were out-of-proportion to the rest of her neurological examination. Remarkably, in the Rey-Osterrieth Complex figure task, she copied with her left hand the outlines of the figure (global aspects), whereas with her right hand she drew the details of that figure (local aspects). While hemispheric lesions have demonstrated single dissociations of spatial processing, these results indicate that a lesion in the corpus callosum can produce a double dissociation for high-level spatial tasks, as local and global spatial perception are further dissociated with handedness. Interestingly, as little as the posterior third of the corpus callosum is required for proper visuospatial information transfer and integration, which provides important insight into the interhemispheric functional anatomy that underlies visuospatial perception.

## 1. Introduction

Cognitive deficits after callosotomy have shed light on the distinct functions of the two cerebral hemispheres [5]. Although there is no simple dichotomy, the right hemisphere primarily attends to the global aspects of spatial processing, while the left hemisphere appears more specialized for the local aspects of space [3,10, 15].

The concept of lateralized spatial processing was deduced from patients with unilateral hemispheric lesions who either showed the ability to perceive the whole image without being aware of its parts [2] or the ability to see details without being aware of the global aspects [9]. These single dissociations of visuospatial perception reflected on lesions of the left and right temporo-parietal cortices that produced deficits in local and global attentional control of visual perception, respectively [2,9,15]. Functional imaging studies have independently demonstrated lateralized activity with global and local spatial tasks [3,10]: the right lingual gyrus and temporoparietal cortex are active during global visual attention, while the left inferior occipital cortex and left temporo-parietal cortex are activated for locally directed visual attention. Interestingly, the temporo-parietal cortex appears to exert attentional control over the prestriate areas and is preferably active during an attentional switch from local to global spatial

ISSN 0953-4180/08/$17.00 © 2008 – IOS Press and the authors. All rights reserved perception (and vice versa), while the prestriate cortex is active during sustained or directed attention.

Despite the understanding of hemispheric input into spatial processing, insight into the areas of the corpus callosum that convey spatial information across the hemispheres has not been as well developed. Due to technical constraints, functional imaging and transcranial magnetic stimulation studies can not currently demonstrate particular areas of the corpus callosum that are involved in specific spatial tasks. Moreover, deficits in high-level spatial task performance in patients with defined lesions of the corpus callosum have also not been reported to date. Here, we report such a spatial task deficit in a patient with a lesion in the posterior corpus callosum. This case sheds light on the interhemispheric component of the functional anatomy of spatial processing.

## 2. Methods

This is a single case report of a patient who underwent careful cognitive evaluation. The principles outlined in the Declaration of Helsinki were followed while interviewing this patient. Neuropsychological test results are presented in table 1, and the corresponding references for the details of each test [1,4,6,7,11, 13,16,17] are separately mentioned in the table.

Administration of the Rey-Osterrieth Complex figure (RCF) test was conducted slightly differently than for routine neuropsychological testing. Rather than having the patient look at the RCF for a standard amount of time and then ask the patient to draw the figure from memory, this patient was given full access to viewing the figure at all times for both 5-minute direct copy trials. Therefore, with our patient, memory testing was minimized, but more focus was placed on the ability to integrate visual information. Given that there are currently no published methods available, which objectively and quantitatively score spatial processing deficits in the RCF task, we opted to graphically display the copying pattern and time course. Although this is a qualitative rating, it does allows for complete disclosure of results and also highlights the copying differences at similar times between the tested hemispheres in this patient.

Norms for neglect tasks were obtained from 57 normal subjects, who had no evidence of stroke or other neurological disorder on MRI, a mean age of 64 $\pm$ 11 years, and a mean duration of education of 13.7 $\pm$ 3.5 years. Performance across the various neglect tests ranged from 0 $\pm$ 0% errors (clock copy) to 3.2 $\pm$ 3.4% errors (line bisection). Critically, as none of the normal subjects made $>$ 10% errors on any of these tests, neglect was defined as $>$ 10% errors (2 standard deviations from the highest mean error rate) on any task.

The following clarifications are provided for how some of the neglect tasks were tested. In the flower copy task, the patient was asked to copy a daisy that consisted of a stem, two leaves, and seven petals, using right and left hand separately. In the line cancellation task, each of 28 pseudo-randomly arranged lines was to be cancelled with only one mark; lines were presented 45$^\circ$ to the left and 45$^\circ$ to the right of the midsagittal plane, as well as at the midsagittal plane of the viewer. In the horizontal reading task, 2 columns of 15 words were read aloud; below the columns, 5 sentences were read aloud. In the vertical reading task, four and five letter words were printed vertically. For visual extinction, the hands of the administrator were placed in the right and left visual field approximately 3 feet from the patient. The patient was asked to look at the administrator's nose while determining if movement was present either unilaterally or bilaterally. The presentation of unilateral and bilateral movements was intermixed. For tactile extinction, testing was performed with the eyes of the patient being closed. Finally, in the gap-detection test, a page of 30 circles was presented, where 10 circles each had a gap on the left side, on the right side, or no gap; two variations of this test were administered, with large (2.8 $\times$ 2.2 cm) and small circles (1.5 $\times$ 1.5 cm), respectively; each circle was marked, using an X for a circle with a gap and circling the circles without gaps; both the percentage of circles unmarked on the left half of the page (a measure of egocentric neglect) and the percentage of left gaps undetected (a measure of allocentric neglect) were counted; the circles with undetected left gaps were determined by being falsely circled by the patient.

## 3. Case results

A 60-year-old-right-handed woman initially presented with symptoms of topographical disorientation, as she was unable to find her way around while driving and then even when being at home. Her bedside neurological exam was remarkable for a complete inability to mimic simple geometric figures constructed by the examiner's hand. Magnetic resonance imaging (MRI) revealed a mass lesion that was highly suggestive of a

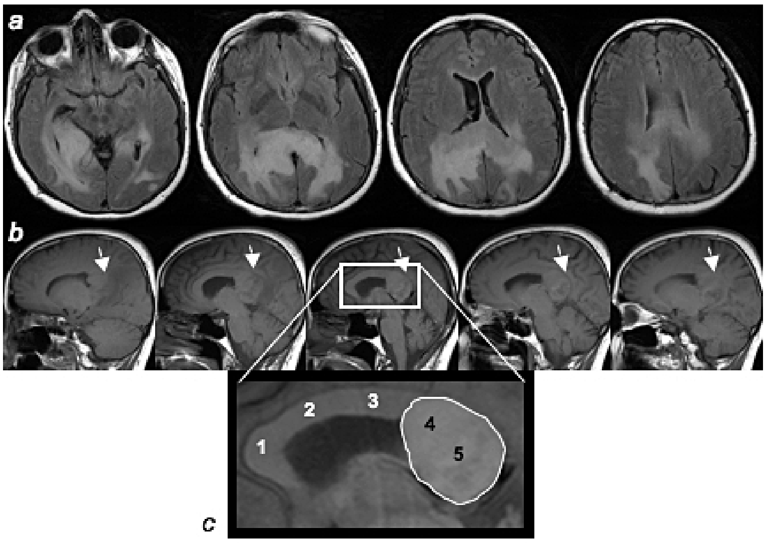

Fig. 1. MRI images of the patient's brain. Axial T2 FLAIR (**a**) and sagittal T1 (**b**) images of mass lesion (arrows) showing primary involvement of the splenium of the corpus callosum. (**c**) Magnification of corpus callosum (mass lesion circled) illustrating the probable disconnected brain regions based upon known location [8] (1. premotor, prefrontal; 2. motor; 3. somatosensory; 4. parietal, temporal, insular; 5. striate, peristriate).

Table 1
Performance on Neuropsychological tests and Unilateral Spatial Neglect Tasks

| *Neuropsychological tests* | *Cognitive function tested* | *Score* |
|---|---|---|
| Mini-Mental State Exam [4]* | specific modalities of mental status | 24/30 |
| Wechsler Memory Scale [16] | | 12/14 |
| Forward Digit span [17] | working memory and attention | 7/16 |
| Backward Digit span [17] | | 4/14 |
| COWA (F-A-S) [1] | word fluency and speed of executive functions | 43 |
| Pyramids and Palmtrees [7] | association of semantically related objects | 51/53 |
| Left-hand apraxia-real objects | | 7/7 |
| Left-hand apraxia-imagined objects** | | 4/4 |
| Hooper [6] | mental reconstruction of puzzle like objects | 13/14 |
| *Spatial neglect tasks* | | |
| Visual Extinction | unilateral extinction | 5/10 |
| Tactile Extinction | (functional hemispheric disconnection) | 10/10 |
| Oral Reading*** | allocentric neglect (stimulus-centered) | 26/30 |
| Vertical Reading | object-centered neglect | 49/52 |
| Oral Spelling | | 36/37 |
| Ogden Scene Right Hand | visual construction and neglect | 31/36 |
| Ogden Scene Left Hand [11] | | 32/36 |
| Copying Flowers (Both Hands) | | 11/11 |
| Line Cancellation | egocentric neglect (viewer-centered) | 84/84 |
| Gap-Detection Test [13] | egocentric and allocentric neglect | 58/60 |

*Deductions for date, hospital floor, delayed object recall, and spelling WORLD backwards (3 errors).
**Correct performance shows that an intact anterior corpus callosum can transfer language information to the right hemisphere.
***All errors were made on the left side of the words, indicating left neglect dyslexia.

tumor and primarily affected the posterior portion of the corpus callosum (splenium), with some infiltration bilaterally into the surrounding white matter (Fig. 1a,b,c). Given the significant visuospatial deficit, a detailed evaluation of cognitive functions was pursued.

The performance was within the normal range across most neuropsychological tests, including tests for apraxia, memory, verbal fluency, semantic relatedness, and visual perceptual integration (Table 1, top half). In tests for spatial neglect, however, some deficits became

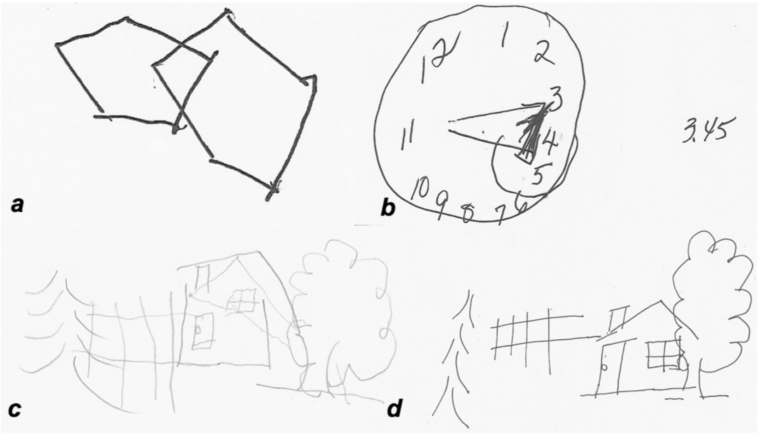

Fig. 2. Spatial construction and neglect tasks. Intersecting pentagons copy (**a**), clock copy (**b**), and drawings of the Ogden scene with left (**c**) and right hand (**d**).

evident (Table 1, bottom half). While tasks such as line cancellation, flower copy, intersecting pentagons (Fig. 2a), and gap-detection were performed well, the patient made 5 errors (30%) when asked to draw a clock with her right hand that depicted the time of 3:45 (Fig. 2b). These errors included incorrect placement of numbers (shift to the right side), and difficulty in placing the minute hand. Interestingly, the patient understood the time concept of 3:45 and could read clocks depicting that time, but she would point the minute hand to the number 4, look confused, then circle the numbers 4 & 5 and state "and this is for the 45". Furthermore, consistent with left-sided processing deficits that were seen with visual extinction, the patient showed neglect dyslexia in the oral reading task that had a clear predilection to the left side of the words only. Finally, the patient made errors when drawing the Ogden scene with either hand (Fig. 2c–d), although these were not clearly errors of spatial neglect. Rather, they were errors of spatial processing, as no bias towards global and local aspects of space or elements of neglect were displayed.

Most remarkable were the findings in tests for visuospatial construction. When the patient was asked to copy the Rey-Osterrieth Complex Figure (complex geometric figure) [12,14], striking deficits were noted that were qualitatively different between both hands. As the patient copied the figure with her right hand (left hemisphere), she used a local approach by drawing the individual elements. The placement and number of local features suggested a disregard for the exact location that these features occupy within the figure and an inability to appreciate previously copied elements (Fig. 3a). In contrast, when subsequently drawing the figure anew with her left hand (right hemisphere), she used a global approach by focusing on the figure outline (Fig. 3b). Furthermore, the patient took a very long time to perform the copy task with each hand. For example, when 150 seconds into the left-hand trial, she ceased copying the figure, compared the RCF to her attempt, stated "I don't know what else to do", and only continued to draw additional figure components at 270 seconds into the trial (Fig. 3b). On the next day, the patient was retested with the Rey- Osterrieth Complex Figure, but the hand order was reversed (left before right). In this manner, we wished to determine whether the observed difference in behavior on the prior day could be explained by "learning" the global aspects of the figure while using the right hand. However, the spatial processing deficits and method of copying persisted.

## 4. Discussion

The performance of this patient solidifies the concept of lateralized spatial processing. By applying a local copying method when responding with her left hemisphere, the patient did not recognize the "forest". Conversely, by showing a consistent global copying strategy when responding with her right hemisphere, the patient failed to see the "trees". Interestingly, these results not merely reflect on a single dissociation state, but on double dissociation: local and global processing was additionally dissociated with handedness. Hence, the left hand executed the global copying approach, while the right hand drew with a local method. This property is attributable to the lesion in the posterior

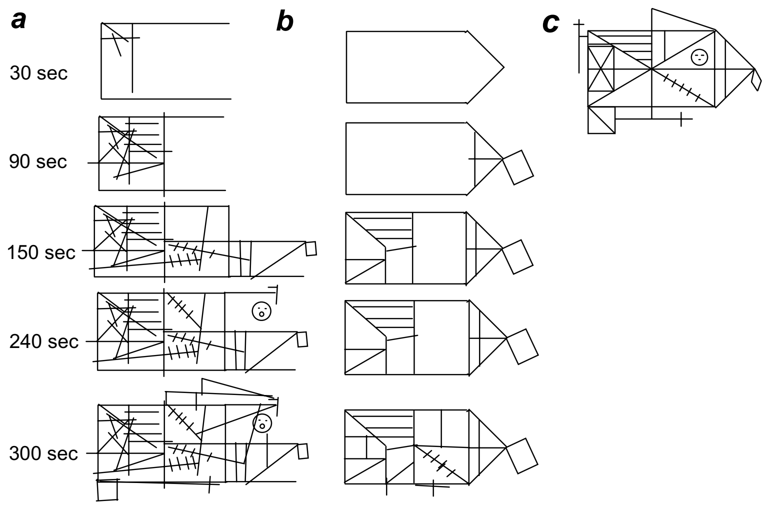

Fig. 3. Sequential drawings of the Rey-Osterrieth Complex figure. (**a**) Right-hand copy demonstrates the patient's inability to comprehend the global form. (**b**) Left-hand copy shows the immediate acquisition of the global form, while local features are absent in the initial 90 seconds. The long pause in drawing right-sided local aspects likely indicates right hemispatial neglect. (**c**) Correct version of Rey-Osterrieth Complex figure.

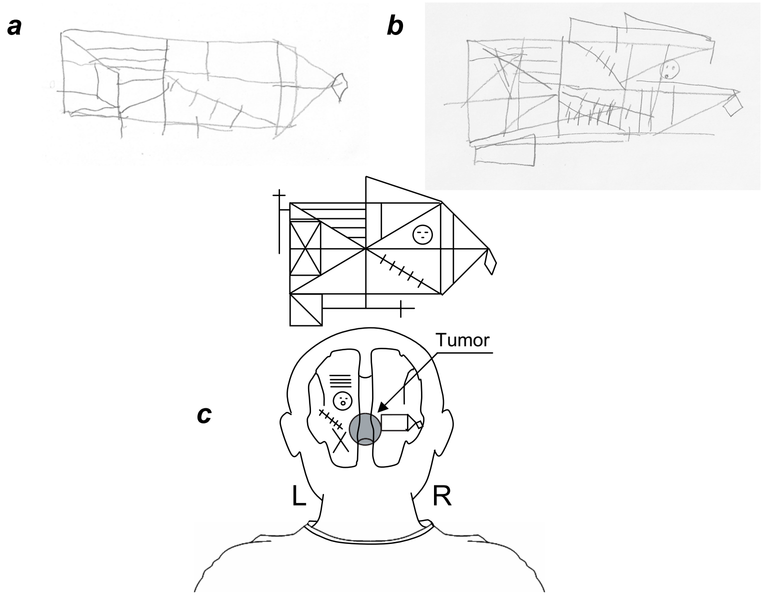

Fig. 4. Final Rey-Osterrieth Complex Figure (RCF) and cognitive model (**a**) Final L-hand RCF copy. (**b**) Final R-hand RCF copy. (**c**) A cognitive model, adapted from Gazzaniga and colleagues, depicts how the segregated visuospatial information is unable to cross the corpus callosum.

corpus callosum, which has disrupted normal transfer and integration of spatial information between the hemispheres. Accordingly, when the disconnected left hemisphere directed the right hand to draw the complex figure, information transfer from the right hemisphere was disrupted, which resulted in global processing deficits (RCF), left neglect (clock copy) and left neglect dyslexia (oral reading). Similarly, when the disconnected right hemisphere directed the left hand to draw the complex figure, the observed local processing deficits and neglect for the right visual field indicate an inability of the left hemisphere to shuttle right-sided and local aspects of spatial information to the right hemisphere (see Fig. 4c for model of the drawing strategy). In addition, consistent with a subcortical process, task performance was very much slowed. Curiously, the processing breakdown in this patient was subtle enough to display a double dissociation only with the most difficult tasks (RCF, clock copy), while in many less complex tasks (Ogden scene, gap detection, line cancellation) each hemisphere was able to perform sufficiently. Although aspects of task demand dependent deficits may be deduced from observed dissociations in case reports [2,3,5,9,10,15], to our knowledge such deficits have not previously been described as a double dissociation for local and global processing in a single patient.

Importantly, this study complements previous lesion studies in defining the functional anatomy of the neuronal circuitry that underlies visuospatial processing. Previous studies have emphasized the importance of hemispheric areas for the differential attentional control on spatial processing, in particular the temporo-parietal cortex [2,9,15]. This study, to our knowledge for the first time, now provides lesional evidence for understanding the interhemispheric cross-talk of global and local spatial information for a high-level spatial task. While the corpus callosum is the obvious anatomical substrate for such cross-talk, it is initially surprising that the double dissociation is manifest in the presence of an intact anterior and middle corpus callosum. However, given the known location of the white matter tracts within the corpus callosum (Fig. 1c), these findings make sense, as the posterior corpus callosum transfers information between the temporo-parietal cortices. The inability of the intact anterior and middle corpus callosum to compensate for the deficits indicates that the posterior corpus callosum is required for accurate global and local spatial processing (visuospatial perception) and execution of a complex demand task (visuospatial performance). Whether the posterior corpus callosum is also sufficient for these functions remains to be investigated. Analysis of complex demand tasks in patients with intact posterior but defective anterior and middle corpus callosum will help to shed light on this question. Notably, such analysis will begin to assess the degree of motor information and visuospatial processing information that is spatially transferred across the distinct regions of the corpus callosum. In this regard, it will be important to determine how much motor and visuospatial information travels across the posterior and anterior-middle corpus callosum, respectively.

## Acknowledgments

We thank our patient for her participation; Brenda Rapp, for teaching J.T.K. the beauty of double dissociations; and Drs. Argye Hillis and Ola Selnes for helpful discussion. This work was supported by NINDS RO1NS47691 (Argye E. Hillis).
